# Dopamine gene methylation patterns are associated with obesity markers and carbohydrate intake

**DOI:** 10.1002/brb3.1017

**Published:** 2018-07-11

**Authors:** Omar Ramos‐Lopez, Jose I. Riezu‐Boj, Fermin I. Milagro, J. Alfredo Martinez, MENA Project, I Abete, AB Crujeiras, M Cuervo, L Goni, A Marti, MA Martinez‐Gonzalez, MJ Moreno‐Aliaga, S Navas‐Carretero, R San‐Cristobal, JL Santos, MA Zulet

**Affiliations:** ^1^ Department of Nutrition, Food Science and Physiology Center for Nutrition Research University of Navarra Pamplona Spain; ^2^ Navarra Institute for Health Research (IdiSNA) Pamplona Spain; ^3^ CIBERobn, Fisiopatología de la Obesidad y la Nutrición Carlos III Health Institute Madrid Spain; ^4^ Madrid Institute of Advanced Studies (IMDEA Food) Madrid Spain

**Keywords:** diet, dopamine, epigenetics, obesity, *SLC18A1*, *SLC6A3*

## Abstract

**Introduction:**

Dopamine (DA) is a neurotransmitter that regulates the rewarding and motivational processes underlying food intake and eating behaviors. This study hypothesized associations of DNA methylation signatures at genes modulating DA signaling with obesity features, metabolic profiles, and dietary intake.

**Methods:**

An adult population within the Methyl Epigenome Network Association project was included (*n* = 473). DNA methylation levels in white blood cells were measured by microarray (450K). Differentially methylated genes were mapped within the dopaminergic synapse pathway using the KEGG reference database (map04728). Subsequently, network enrichment analyses were run in the pathDIP portal. Associations of methylation patterns with anthropometric markers of general (BMI) and abdominal obesity (waist circumference), the blood metabolic profile, and daily dietary intakes were screened.

**Results:**

After applying a correction for multiple comparisons, 12 CpG sites were strongly associated (*p *<* *0.0001) with BMI: cg03489495 (*ITPR3*), cg22851378 (*PPP2R2D*), cg04021127 (*PPP2R2D*), cg22441882 (*SLC18A1*), cg03045635 (*DRD5*), cg23341970 (*ITPR2*), cg13051970 (*DDC*), cg08943004 (*SLC6A3*), cg20557710 (*CACNA1C*), cg24085522 (*GNAL*), cg16846691 (*ITPR2*), and cg09691393 (*SLC6A3*). Moreover, average methylation levels of these genes differed according to the presence or absence of abdominal obesity. Pathway analyses revealed a statistically significant contribution of the aforementioned genes to dopaminergic synapse transmission (*p *=* *4.78E−08). Furthermore, *SLC18A1* and *SLC6A3* gene methylation signatures correlated with total energy (*p *<* *0.001) and carbohydrate (*p *<* *0.001) intakes.

**Conclusions:**

The results of this investigation reveal that methylation status on DA signaling genes may underlie epigenetic mechanisms contributing to carbohydrate and calorie consumption and fat deposition.

## 1 INTRODUCTION

Besides homeostatic processes concerning energy and nutrient metabolic control, eating behavior is also regulated by hedonic (nonhomeostatic) mechanisms (Hernández Ruiz de Eguilaz et al., 2018), which are thought to be driven by the rewarding properties of foods and specific nutritional and behavioral afferent signals (Ziauddeen, Alonso‐Alonso, Hill, Kelley, & Khan, 2015). In this context, it has been reported that similar to alcohol and other drugs of abuse, highly palatable foods (rich in sugars and fat) can trigger neuroadaptive responses in brain reward circuitries (Alonso‐Alonso et al., 2015). These effects can stimulate feeding behavior and related attitudes independent of energy status or overcome other signals of satiety and hunger, contributing to overeating and weight gain (Kenny, 2011). Because of the rising prevalence of obesity and the widespread availability of calorie‐dense foods, understanding the hedonic processes underlying food consumption and behavioral cues beyond metabolic needs has become a priority in obesity research (Stice, Figlewicz, Gosnell, Levine, & Pratt, 2013).

Reward and gratification associated with palatable food consumption are partially mediated by abrupt dopamine (DA) increases in the nucleus accumbens and the ventral tegmental area (Singh, 2014). Moreover, the amount of DA released after consuming a preferred meal eventually correlates with the degree of experienced pleasure (Small, Jones‐Gotman, & Dagher, 2003). Thus, disruption of DA activity can lead to loss of control over intake and continued consumption despite negative consequences, being both behaviors commonly seen in addiction and obesity (Volkow, Wang, Tomasi, & Baler, 2013). Consistently, deficits in mesolimbic DA neurotransmission have been linked to diet‐induced obesity in rats (Geiger et al., 2009). In humans, imaging studies suggest that obese subjects may suffer impairments in dopaminergic pathways involved in reward sensitivity, incentive motivation, conditioning, and control (Volkow, Wang, Fowler, Tomasi, & Baler, 2012). Therefore, some novel strategies in the prevention and treatment of obesity target to manage DA functions (Blum et al., 2018).

Emerging evidences suggest that several genetic and epigenetic factors modulate the relationships between DA signaling, overconsumption, and obesity (Blum, Thanos, & Gold, 2014; Stice, Yokum, Zald, & Dagher, 2011). For instance, polymorphisms near or within key genes regulating dopaminergic synapse, including catechol‐o‐methyltransferase (*COMT*), D2 receptor (*DRD2*), and DA active transporter (*DAT*,* SLC6A3*) have been associated with altered reward circuitry responsivity related to a spectrum of addictive behaviors (Stice et al., 2011). Moreover, differential DNA methylation patterns at *DAT* and tyrosine hydroxylase (*TH*) were linked to altered DA‐related gene expression in response to chronic intake of high‐fat diet in mice (Vucetic, Carlin, Totoki, & Reyes, 2012). Furthermore, a set of transcriptional and epigenetic changes in the hypothalamus of prenatally stressed female rats were implicated in an increased susceptibility to a high‐fat‐sucrose diet (Paternain et al., 2012). This study hypothesized associations of DNA methylation signatures at genes modulating DA signaling with obesity features and accompanying metabolic profiles as well as an epigenetic influence on macronutrient intake.

## 2 MATERIALS AND METHODS

### 2.1 Subjects

A transversal nutriepigenomic analysis was conducted in a general adult population within the Methyl Epigenome Network Association (MENA) project (*n* = 473). The MENA cohort is constituted by previous clinical trials analyzing genome‐environmental interactions concerning weight management and associated metabolic outcomes (Abete et al., 2015; Huerta, Navas‐Carretero, Prieto‐Hontoria, Martínez, & Moreno‐Aliaga, 2015; Larsen et al., 2010; Martínez‐González et al., 2014; Petersen et al., 2006; San‐Cristobal et al., 2015; Santos et al., 2016; Zulet et al., 2011). Each study received ethical approval from appropriate local Human Research Ethics Committees. In addition, all procedures carried out throughout this investigation were in agreement with the ethical principles of the 2013 Helsinki Declaration (World Medical Association, 2013). Also, subject's information was coded to insure full anonymity. All participants gave their informed consent before inclusion in the study.

### 2.2 Anthropometric measurements and blood pressure

Anthropometric measurements including weight, height, and waist circumference (WC) were collected by trained health personnel using conventional methods (de la Iglesia et al., 2014; Mansego, Milagro, Zulet, & Martinez, 2015). Body mass index (BMI) was calculated dividing weight (kg) by squared height (m^2^). The World Health Organization (2017) classification of BMI in adults was used to characterize normal weight (BMI 18.5–24.9 kg/m^2^) and overweight/obese individuals (BMI ≥25 kg/m^2^). Abdominal obesity (AO) was defined based on established WC cutoffs for men (>102 cm) and women (>88 cm) as reported by the National Cholesterol Education Program (2002). Systolic blood pressure (SBP) and diastolic blood pressure (DBP) were measured from the right arm of each participant with a sphygmomanometer after a 15‐min rest. The average of two successful readings was recorded following the World Health Organization criteria (2004) (Whitworth, & Chalmers, 2004).

### 2.3 Biochemical tests

Venous blood samples were drawn from each participant by venipuncture after a 12‐hr overnight fast. Glucose, total cholesterol (TC), high‐density lipoprotein cholesterol (HDL‐c), and triglycerides were determined in the automatic analyzer Pentra C200 (HORIBA Medical, Madrid, Spain) with appropriate commercial kits provided by this company. Low‐density lipoprotein cholesterol was calculated using the Friedewald equation: LDL‐c = TC − HDL‐c − triglycerides/5 as described elsewhere (Ramos‐Lopez et al., 2018b). Plasma concentrations of insulin (Mercodia, Uppsala, Sweden) were measured using specific enzyme‐linked immunosorbent assays and assessed by means of an automated analyzer system (Triturus, Grifols, Barcelona, Spain). Insulin resistance was estimated by the homeostatic model assessment‐insulin resistance (HOMA‐IR) index according to the following formula: (fasting insulin (mU/L) × plasma glucose (mmol/L)/22.5) as previously reported (Crujeiras et al., 2014). Triglyceride‐glucose (TyG) index was calculated as: (ln [fasting triglycerides (mg/dl) × fasting plasma glucose (mg/dl)/2]) as described elsewhere (Navarro‐González, Sánchez‐Íñigo, Pastrana‐Delgado, Fernández‐Montero, & Martinez, 2016).

### 2.4 Dietary assessment

Dietary data were additionally obtained from 247 subjects of the MENA cohort, which presented similar characteristics regarding the whole population. The habitual consumption of 137 food items during the previous year was evaluated with a validated, semiquantitative food frequency questionnaire (de la Fuente‐Arrillaga, Ruiz, Bes‐Rastrollo, Sampson, & Martinez‐González, 2010). Food frequencies (daily, weekly, monthly or never), portions, and serving sizes were computed and further converted to daily energy (kcal) and macronutrient intakes (g) using recognized Spanish food composition tables, as described elsewhere (Goni, Aray, Martínez, & Cuervo, 2016). Nutrients from the diet (carbohydrates, protein, and fat) were adjusted by total energy intake using the residual method, as previously reported (Carraro et al., 2016).

### 2.5 DNA methylation analyses

Blood samples were centrifuged (2,000 g, at 4°C for 15 min) to isolate white blood cells (WBCs) from whole blood. WBCs were immediately frozen at −80°C in buffy coat until use as described elsewhere (Arpón et al., 2016). Genomic DNA was extracted from WBC using the Master Pure DNA purification kit (Epicentre Biotechnologies, Madison, WI, USA) following instructions provided by the supplier. DNA quality was assessed with the PicoGreen^®^ dsDNA Quantitation Reagent (Invitrogen, Carlsbad, CA, USA). A total of 500 ng of purified DNA was treated with sodium‐bisulfite using the EZ‐96 DNA Methylation Kit (Zymo Research Corporation, Irvine, CA, USA) according to the manufacturer's protocol. Modified DNA samples were whole‐genome amplified and hybridized to Infinium Human Methylation 450K BeadChips (Illumina, San Diego, CA, USA) as detailed elsewhere (Mansego, Garcia‐Lacarte, Milagro, Marti, & Martinez, 2017). The scanning of the samples was carried out with the Illumina HiScanSQ system, and the image intensities were extracted with the GenomeStudio Methylation Software Module, v1.9 (Illumina).

DNA methylation data preprocessing has been recently described (Ramos‐Lopez, Riezu‐Boj, Milagro, & Martinez, 2018c; Ramos‐Lopez et al., 2018a). Briefly, CpG methylation levels were expressed as β values, which are calculated as the ratio between the Illumina methylated probe intensities and the overall probe intensities (sum of methylated and unmethylated probe intensities). β values ranging from 0 (unmethylated) to 1 (completely methylated) were used, as previously reported (Weinhold, Wahl, Pechlivanis, Hoffmann, & Schmid, 2016). Methylation data were peak‐based corrected for type I and type II bias and subsequently normalized using a categorical Subset Quantile Normalization method (Touleimat & Tost, 2012). Probes containing single nucleotide polymorphisms, those hybridizing to multiple genomic locations, or associated with X and Y chromosomes, were removed (Naeem et al., 2014; Nordlund et al., 2013). The ComBat normalization method was applied to adjusting for nonbiological experimental variation (Johnson, Li, & Rabinovic, 2007). Moreover, an additional analysis to estimate the variation explained due to different cell subtypes (granulocytes, monocytes, B cells, T cells‐CD8^+^, T cells‐CD4^+^, and natural killer cells) was performed according to the Houseman criteria (Houseman et al., 2012).

### 2.6 Pathway analyses

To test the hypothesis of this study, differentially methylated genes were mapped to the dopaminergic synapse pathway (map04728) using the online Kyoto Encyclopedia of Genes and Genomes (KEGG) reference database (http://www.genome.jp/kegg/pathway.html). The Pathway Data Integration Portal (pathDIP) platform (http://ophid.utoronto.ca/pathdip/) was used to perform pathway enrichment analyses, with a confidence level of 99%. *p* value corresponding to KEGG source was then reported.

### 2.7 Statistical analyses

The Kolmogorov–Smirnov test was used to determine data distribution. All study variables were normally distributed (*p *>* *0.05). Results are expressed as means ± standard deviations (*SD*), meanwhile, men and women are presented as number of cases. Statistical differences between AO groups were analyzed by student *t* test (continuous variables) and chi‐square test (dichotomous variables). A linear regression model concerning BMI outcomes was computed using the LIMMA package for R software, which was adjusted by covariates such as age, sex, study cohorts, and DNA methylation chips. The Benjamini–Hochberg correction for multiple comparisons was applied. Statistically significant thresholds were based on False Discovery Rate (FDR) cutoffs (*p *<* *0.05) and B‐statistic values from LIMMA (B > 0). The LIMMA B‐statistic is the log‐odds that a determined gene is differentially methylated. The cutoff B value above 0 implies that a CpG is more likely to be differentially methylated than to not be differentially methylated, giving a reasonable balance of false positives and false negatives (Yang et al., 2011). Best BMI‐associated CpGs were selected according to stricter FDR values (*p *<* *0.0001). Further linear regression analyses adjusted by age and sex were performed to evaluate associations of methylation values at DA signaling genes with anthropometric measurements, the metabolic profile, and dietary intakes. *p* < 0.05 was considered statistically significant. Statistical analyses were performed in the IBM SPSS software version 20 for Windows (IBM Inc., Armonk, NY, USA). GraphPad Prism^®^ program version 6.0C (La Jolla, CA, USA) was used to graphically illustrate significant correlations.

## 3 RESULTS

Demographic, anthropometric, and metabolic characteristics as well as dietary intake of the study population categorized by the presence or absence of AO are reported (Table 1). About 82% of the study population presented excessive body weight according to the BMI classification of the World Health Organization (BMI ≥25 kg/m^2^). Moreover, 57% of the whole sample presented AO based on WC values. No differences between AO groups concerning age and sex were found. Subjects with AO had statistically significant higher levels of blood pressure, insulin, HOMA‐IR, TyG index, and worse lipid profile as well as greater daily dietary consumption of calories, carbohydrates, protein, and fat compared to non‐AO individuals.

**Table 1:** Demographic, anthropometric, and metabolic characteristics as well as dietary intake of the study population categorized by the presence or absence of abdominal obesity

Variable	Non‐AO	AO	*p* value
*n*	205	268	—
Age (years)	46.0 ± 17.7	47.8 ± 11.0	0.182
Men/women	83/122	87/181	0.082
Anthropometric and clinical data			
Weight (kg)	68.2 ± 10.7	91.9 ± 17.8	<0.001
BMI (kg/m^2^)	25.5 ± 3.2	33.5 ± 4.6	<0.001
WC (cm)	83.1 ± 11.2	105.5 ± 11.9	<0.001
SBP (mmHg)	121.5 ± 36.4	106.8 ± 42.2	<0.001
DBP (mmHg)	75.1 ± 22.1	89.3 ± 38.2	<0.001
Metabolic profile			
Glucose (mg/dl)	99.4 ± 33.4	104.4 ± 26.6	0.080
Insulin (mIU/L)	6.8 ± 3.7	11.1 ± 7.8	<0.001
HOMA‐IR index	1.38 ± 0.84	2.99 ± 2.59	<0.001
TC (mg/dl)	197.7 ± 40.3	210.0 ± 39.6	0.001
HDL‐c (mg/dl)	57.4 ± 13.5	50.7 ± 12.8	<0.001
LDL‐c (mg/dl)	118.6 ± 38.2	134.4 ± 34.2	<0.001
TG (mg/dl)	111.4 ± 62.7	125.4 ± 77.3	0.041
TyG index	4.57 ± 0.31	4.65 ± 0.33	0.015
Dietary intake			
Energy (Kcal/day)	2,373 ± 508	2,679 ± 844	0.003
Carbohydrates (g/day)	239.6 ± 68.1	272.1 ± 107.3	0.013
Protein (g/day)	93.2 ± 18.6	109.5 ± 30.7	<0.001
Fat (g/day)	107.3 ± 22.6	119.5 ± 41.2	0.012

*Note*

Continuous variables are represented as means ± standard deviations. Men and women are number of cases. AO: abdominal obesity; BMI: body mass index; DBP: diastolic blood pressure; HDL‐c: high‐density lipoprotein cholesterol; HOMA‐IR index: homeostatic model assessment‐insulin resistance index; LDL‐c: low‐density lipoprotein cholesterol; SBP: systolic blood pressure; TC: total cholesterol; TG: triglycerides; TyG index: triglyceride‐glucose index; WC: waist circumference. Dietary intake was available from 247 subjects.

Overall, 119 CpG sites at genes integrating the dopaminergic synapse pathway correlated with BMI (kg/m^2^). Of these, 44 CpGs showed best associations (*p *<* *0.0001). After adjusting by age plus sex and the appropriate correction for multiple comparisons, 12 CpGs at 9 genes remained statistically significant: cg03489495 (*ITPR3*), cg22851378 (*PPP2R2D*), cg04021127 (*PPP2R2D*), cg22441882 (*SLC18A1*), cg03045635 (*DRD5*), cg23341970 (*ITPR2*), cg13051970 (*DDC*), cg08943004 (*SLC6A3*), cg20557710 (*CACNA1C*), cg24085522 (*GNAL*), cg16846691 (*ITPR2*), and cg09691393 (*SLC6A3*). Genomic and statistical data of these CpG sites sorted by FDR values are presented (Table 2). Most of them are located in coding (*n* = 5) and promoter (*n* = 4) regions, meanwhile, the rest is mapped within untranslated trailers (*n* = 3).

**Table 2:** Genomic and statistical data of CpG sites at dopamine pathway genes statistically associated with BMI

CpG_ID^a^	Illumina_ID	Gene name	Gene symbol	CHR position^b^	Genomic region	*p* value	FDR	B	*r* ^2^
1	cg03489495	Inositol 1,4,5‐trisphosphate receptor type 3	*ITPR3*	6:33,588,875	Body	2.5E−14	9.2E−11	20.82	0.105
2	cg22851378	Protein phosphatase 2 regulatory subunit Bdelta	*PPP2R2D*	10:133,747,932	Body	2.4E−09	3.9E−07	9.55	0.072
3	cg04021127	Protein phosphatase 2 regulatory subunit Bdelta	*PPP2R2D*	10:133,747,926	TSS1500	1.6E−08	1.6E−06	7.67	0.059
4	cg22441882	Solute carrier family 18 member A1	*SLC18A1*	8:20,040,654	3′UTR	3.7E−08	2.9E−06	6.87	0.054
5	cg03045635	Dopamine receptor D5	*DRD5*	4:9,783,198	5′UTR	2.6E−07	1.2E−05	4.98	0.079
6	cg23341970	Inositol 1,4,5‐trisphosphate receptor type 2	*ITPR2*	12:26,782,390	TSS1500	4.6E−07	1.8E−05	4.42	0.055
7	cg13051970	Dopa decarboxylase	*DDC*	7:50,628,968	Body	4.7E−07	1.8E−05	4.42	0.045
8	cg08943004	Solute carrier family 6 member 3	*SLC6A3*	5:1,416,873	1stExon	4.8E−07	1.9E−05	4.38	0.050
9	cg20557710	Calcium voltage‐gated channel subunit alpha1 C	*CACNA1C*	12:2,788,782	Body	5.8E−07	2.1E−05	4.20	0.052
10	cg24085522	G protein subunit alpha L	*GNAL*	18:11,849,055	3′UTR	7.4E−07	2.5E−05	3.97	0.042
11	cg16846691	Inositol 1,4,5‐trisphosphate receptor type 2	*ITPR2*	12:26,986,520	TSS1500	2.3E−06	5.8E−05	2.87	0.067
12	cg09691393	Solute carrier family 6 member 3	*SLC6A3*	5:1,417,003	TSS1500	2.5E−14	9.8E−05	2.16	0.034

*Notes*

Data are sorted by FDR values.

B: LIMMA B‐statistic from LIMMA; BMI: body mass index; CHR: chromosome; FDR: False Discovery Rate.

^a^Studied CpG identifier. ^b^CpG locations were mapped using GRCh37 version of the genome from Ensembl platform.

**Figure 1:**
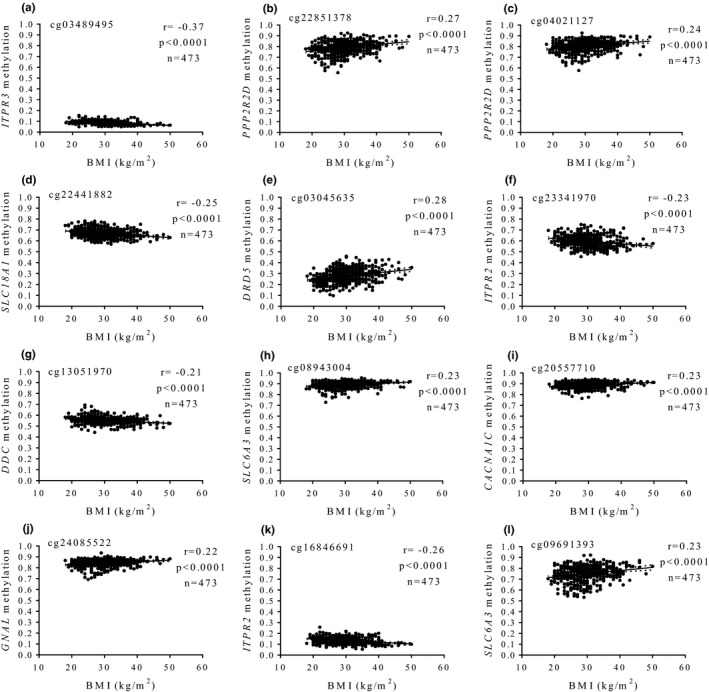
Associations between methylation levels (beta values) at dopamine pathway genes and BMI values. (a) cg03489495, *ITPR3*, (b) cg22851378, *PPP2R2D*, (c) cg04021127, *PPP2R2D*, (d) cg22441882, *SLC18A1*, (e) cg03045635, *DRD5*, (f) cg23341970, *ITPR2*, (g) cg13051970, *DDC*, (h) cg08943004, *SLC6A3*, (i) cg20557710, *CACNA1C*, (j) cg24085522, *GNAL*, (k) cg16846691, *ITPR2*, (l) cg09691393, *SLC6A3*

**Figure 2:**
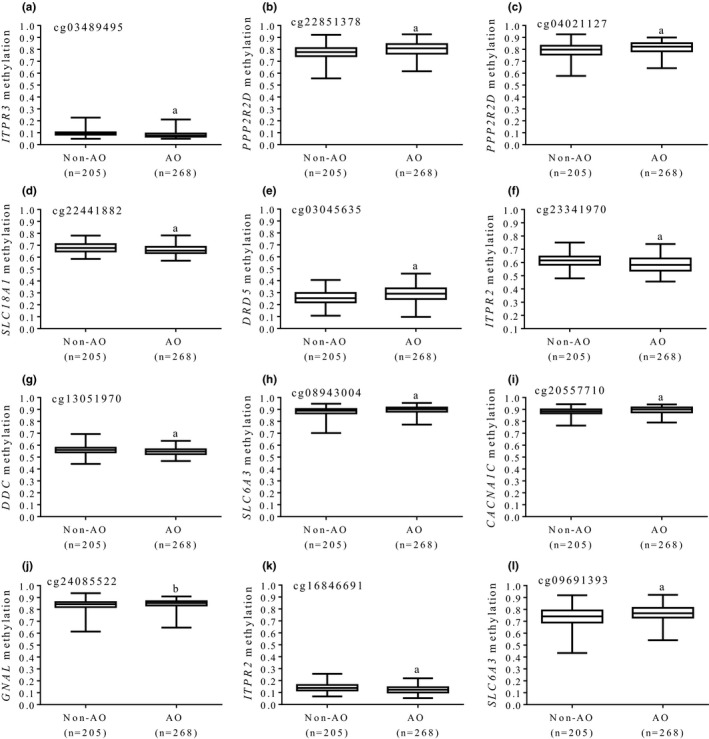
Average methylation levels (beta values) at dopamine pathway genes according to the presence or absence of abdominal obesity. (a) cg03489495, *ITPR3*, (b) cg22851378, *PPP2R2D*, (c) cg04021127, *PPP2R2D*, (d) cg22441882, *SLC18A1*, (e) cg03045635, *DRD5*, (f) cg23341970, *ITPR2*, (g) cg13051970, *DDC*, (h) cg08943004, *SLC6A3*, (i) cg20557710, *CACNA1C*, (j) cg24085522, *GNAL*, (k) cg16846691, *ITPR2*, (l) cg09691393, *SLC6A3*. AO: abdominal obesity. ^a^ *p *<* *0.0001; ^b^ *p *<* *0.001

In a multiple regression model, methylation signatures of the aforementioned 12 CpG sites accounted for about 21% of the variability in BMI (adj. *r* ^2^ = 0.207, *p *<* *0.001). Statistically relevant associations between methylation status and BMI are plotted (Figure 1). Of note, seven CpG sites positively correlated with BMI values, whereas in the remaining analyzed CpGs, negative correlations were found (*n* = 5). Moreover, average methylation levels of each CpG differed according to the presence or absence of AO (Figure 2), with a robust level of significance in most cases (*p *<* *0.0001). No statistically significant relationships between methylation patterns at DA signaling genes with serum levels of glucose, insulin, lipid profile, or blood pressure were detected.

**Figure 3:**
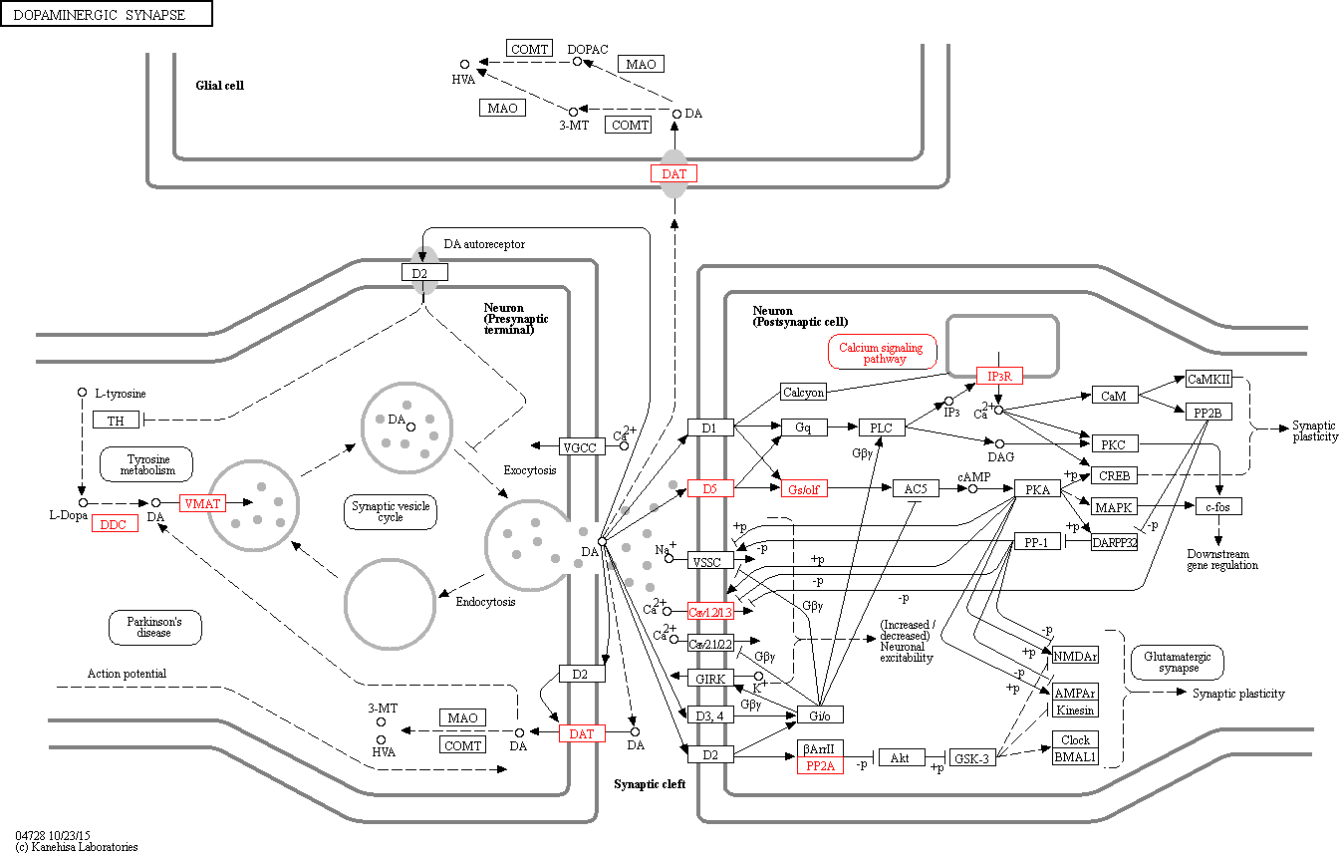
Mapping of BMI‐associated genes within the dopaminergic synapse pathway (red boxes). The following genes were computed: *ITPR3*,* PPP2R2D*,* SLC18A1*,* DRD5*,* ITPR2*,* DDC*,* SLC6A3*,* CACNA1C*,* GNAL*. Figure taken from KEGG reference database (map04728). Pathway enrichment analyses, based on pathDIP (*p *=* *4.78E−08)

Pathway mapping of the BMI‐associated genes within the DA signaling cascade is shown (Figure 3). Interestingly, pathway enrichment analyses revealed a significant contribution of BMI‐associated genes to dopaminergic synapse transmission (*p *=* *4.78E−08), involving complex interactions between presynaptic and postsynaptic cells (Figure 3). These genes modulated key processes involving physiological DA actions such as transport, uptake/reuptake, covalent modifications, and appropriate downstream signal flow.

**Figure 4:**
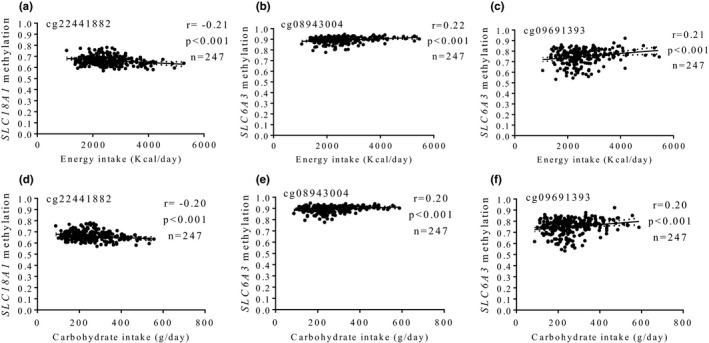
Associations between methylation levels (beta values) at dopamine signaling genes and energy (a–c) and carbohydrate (d–f) intakes. (a, d) cg22441882, *SLC18A1* (b, e) cg08943004, *SLC6A3* (c, f) cg09691393, *SLC6A3*. AO: abdominal obesity

Furthermore, potential associations between DA gene methylation profiles and available daily dietary intakes were evaluated in 247 subjects of the MENA cohort (Figure 4). Thus, methylation at cg22441882 (*SLC18A1*), cg08943004 (*SLC6A3*), and cg09691393 (*SLC6A3*) consistently correlated with total energy consumption (*p *<* *0.001) and carbohydrate intake (*p *<* *0.001). However, no relationships between methylation patterns of these CpG sites and protein or fat intakes were found.

## 4 DISCUSSION

DA is a major (nonhomeostatic) regulator of food intake behaviors (Alonso‐Alonso et al., 2015). In agreement with our hypothesis, the present investigation evidenced associations of DA gene methylation patterns with BMI, AO, and carbohydrate intake, which might serve as epigenetic biomarkers of feeding behavior attitudes, excessive adiposity, and fat deposition. These results are consistent with the fact that disruptions in dopaminergic synapse may lead to overconsumption by altering the rewarding effects elicited by palatable foods (Ziauddeen et al., 2015). In this sense, it has been reported that high‐carbohydrate diets can trigger addictive‐like neurochemical and behavioral responses in vulnerable individuals, contributing to weight gain (Lennerz & Lennerz, 2018). The link between body weight regulation and fat storage and dopaminergic signaling may also rely on the endocrine effects of DA in peripheral tissues such as insulin secretion and specific actions on adipocytes (Rubí & Maechler, 2010). Furthermore, human adipose cells express DA receptors during adipogenesis, suggesting a controlling role of DA in adipose tissue processes (Borcherding et al., 2011).

DA is synthesized through DOPA decarboxylase (DDC) activity and subsequently packed into synaptic vesicles via the SLC18 family of transporter proteins including VMAT1 (SLC18A1) (Lawal & Krantz, 2013). In this study, both *DDC* and *SLC18A1* gene methylation levels negatively correlated with BMI and were downregulated under AO conditions. In addition, a negative correlation between *SLC18A1* methylation and carbohydrate intake was found. Interestingly, decreased AADC activity has been reported in obese mice fed a high‐fat high‐simple‐carbohydrate diet (Moreira‐Rodrigues et al., 2012). Moreover, genome wide and candidate gene studies identified *SLC18A1* as one potential pleiotropic gene overlapped between mood disorders and cardiometabolic diseases (Amare, Schubert, Klingler‐Hoffmann, Cohen‐Woods, & Baune, 2017). Also, a genetic variation in *SLC18A1* made statistically significant contributions to BMI in Chinese subjects (Chen et al., 2013).

Once released from presynaptic axonal terminals, DA interacts with at least five distinct, but closely related G protein‐coupled receptor subtypes (D1 to D5) in the postsynaptic cells, which regulate the physiological actions of DA (Beaulieu, Espinoza, & Gainetdinov, 2015). In particular, the DA receptor D5 (DRD5) belongs to the D1‐class receptors, whose activation stimulates cAMP production by adenylyl cyclase on DA‐receptive cells (Beaulieu et al., 2015). Here, *DRD5* methylation levels positively correlated with BMI and differed according to AO. In a previous work, it was shown that peripheral blood mononuclear cells from individuals presenting AO expressed lower *DRD5* levels compared to subjects without AO (Leite, Lima, Marino, Cosentino, & Ribeiro, 2016). Furthermore, *DRD5* expression negatively correlated with weight, BMI, and WC values, suggesting that AO is associated with downregulation of dopaminergic pathways in blood cells.

The regulation of synaptic and extrasynaptic DA concentrations is an important process that contributes to efficient DA neurotransmission and compartmentalization (Lohr, Masoud, Salahpour, & Miller, 2017). This function is driven by the DA transporter (DAT, SLC6A3), a membrane protein located perisynaptically, where it rapidly recaptures and transports DA from the extracellular space into the cytosol of the presynaptic neuron (Sotnikova, Beaulieu, Gainetdinov, & Caron, 2006). In this study, two CpG sites at *SLC6A3* gene correlated with BMI and carbohydrate intake with a positive trend. Consistently, it was reported that hypothalamic *SLC6A3* was hypermethylated in the promoter region in response to high‐fat‐sucrose diet in prenatally stressed female adult rats (Paternain et al., 2012). Similarly, a significant increase in DNA methylation within the promoter region of *SLC6A3* was found in the ventral tegmental area of mice fed a high‐fat diet, which associated with repressed expression (Vucetic et al., 2012). In humans, methylation changes at the *SLC6A3* gene have been related to prematurity, a known risk factor for obesity (Arpón et al., 2018). Also, *SLC6A3* gene polymorphisms were associated with palatable food intake and WC in children in early stages of development (Fontana et al., 2015). Additionally, genetic variants in *SLC6A3* have been associated with obesity risk in some populations (Bieliński et al., 2017; González‐Giraldo, Trujillo, & Forero, 2017).

Regarding DA‐evoked downstream transducers, different methylation patterns at *ITPR3*,* PPP2R2D*,* ITPR2*,* CACNA1C*, and *GNAL* genes were found to be associated with BMI and AO in this research. According to our results, it has been proposed that a mutation in *Itpr3* gene could influences food choice by impairing the detection of nutrients in mice (Tordoff, Jaji, Marks, & Ellis, 2012). Likewise, a genetic variant in *ITPR3* gene was related to the linking for particular foods in a Silk Road population (Pirastu et al., 2012). Meanwhile, *ITPR2* and *CACNA1C* have been identified as candidate genes associated with addictive tendencies toward food (Pedram, Zhai, Gulliver, Zhang, & Sun, 2017). Of note, *CACNA1C* methylation levels (a concomitant taste signaling molecule) were previously associated with BMI in an adult population (Ramos‐Lopez et al., 2018a, 2018b). Also, a linkage between *ITPR2* locus and central adiposity was reported (Graff et al., 2013; Liu et al., 2014). Until now, there is no evidence showing potential relationships between *PPP2R2D* and *GNAL* genes and obesity.

The strengths of this investigation include a relatively large sample analyzed, and the analysis of DNA methylation status at all genes integrating the dopaminergic synapse pathway. In addition, several potential confounding factors were considered in the methylation‐related statistical analyses such as sex, age, study cohorts, methylation chips, cell subtypes, nonbiological experimental variation, as well as multiple comparison correction. On the other hand, a limitation of this investigation was the lack of expression assays, but RNA samples were not available. This drawback makes it difficult to predict the effects on gene expression of methylation signatures and phenotypic impact, especially CpGs located in nonpromoter regions or those with small changes when comparing AO groups. For example, although the mean methylation levels at cg03489495 (*ITPR3*) statistically differed between non‐AO and AO individuals, it represented approximately a 2% difference in methylation status. Additionally, type I and type II bias cannot be completely ruled out despite of appropriate statistical settings. Of note, some obtained relevant data could have also been lost because of using robust FDR values to select best BMI‐associated CpG sites in the regression analyses.

Another point to comment is the measurement of DNA methylation signatures in peripheral WBC as surrogate of brain cells methylome profiles. Although previous studies support tissue‐specific DNA methylation patterns (Lokk et al., 2014), there is growing evidence in humans suggesting that some methylation marks detected in leukocytes can be reflected in other target tissues, including oral mucosa (San‐Cristobal et al., 2016) and subcutaneous adipose tissue (Crujeiras et al., 2017). Also, homologies between genomic signatures (including DNA methylation patterns) from blood and brain were reported in a rodent model of concussive injury (Meng et al., 2017). Moreover, it has been shown that in addition to human brain, main DA signaling genes are also expressed in circulating human blood cells, including *DDC* (Kokkinou, Nikolouzou, Hatzimanolis, Fragoulis, & Vassilacopoulou, 2009), *SLC18A1* (Amenta et al., 2001), *SLC6A3* (Mill, Asherson, Browes, D'Souza, & Craig, 2002), and *DRD5* (Leite et al., 2016).

Indeed, epigenetic phenomena are important regulators of genome expression and function, which have an impact on diverse physiological and behavioral processes related to food intake, and energy homeostasis (Milagro, Mansego, De Miguel, & Martínez, 2013). Not surprisingly, many epigenetic mechanisms can be implicated in the development of excessive adiposity and associated metabolic risk, including those affecting DA function (Martínez, Milagro, Claycombe, & Schalinske, 2014). In this context, epigenetic modifications at genes involved in DA signaling transmission may help to explain putative relationships between brain reward circuitries, eating behaviors, and body weight status. This knowledge may also be useful for individual disease risk prediction, the search for therapeutic targets, and the design/implementation of nutriepigenomic strategies aimed to prevention, prognosis, and integral management of obesity and accompanying metabolic complications (Ramos‐Lopez et al., 2017).

In conclusion, the results of this investigation reveal that methylation status of DA signaling genes may be one epigenetic regulator contributing to carbohydrate and calorie consumption and obesity development.

## CONFLICT OF INTEREST

The authors declare that there is no conflict of interest concerning this research.

## APPENDIX 1

### 1.1

Other members of the MENA project in alphabetical order are: I Abete, AB Crujeiras, M Cuervo, L Goni, A Marti, MA Martinez‐Gonzalez, MJ Moreno‐Aliaga, S Navas‐Carretero, R San‐Cristobal, JL Santos, and MA Zulet.
